# The effect of culturally tailored continuity of midwifery care on perinatal outcomes for women having a first Nations baby in Victoria, Australia: a prospective non-randomised translational study

**DOI:** 10.1016/j.eclinm.2026.104028

**Published:** 2026-06-24

**Authors:** Della A. Forster, Touran Shafiei, Fiona E. McLardie-Hore, Res McCalman, Gina Bundle, Michelle Newton, Rebecca Hyde, Robyn Matthews, Susan E. Jacobs, Cath Chamberlain, Sue Kildea, Tanisha Springall, Marika Jackomos, Jennifer Browne, Karyn Ferguson, Ngaree Blow, Jane Freemantle, Helen L. McLachlan

**Affiliations:** a Judith Lumley Centre, La Trobe University, Bundoora, Victoria, 3086, Australia; b School of Nursing and Midwifery, La Trobe University, Bundoora, Victoria, 3086, Australia; c The Royal Women’s Hospital, Parkville, Victoria, 3052, Australia; d Molly Wardaguga Research Institute for First Nations Birth Rights, Faculty of Health, Charles Darwin University, Brinken, Northern Territory, 0810, Australia; e Indigenous Health Equity Unit, Melbourne School of Population and Global Health, University of Melbourne, Carlton, Victoria, 3053, Australia; f School of Nursing and Midwifery, Griffith University, Meadowbrook, Queensland, 4131, Australia; g First Peoples Strategy and Advice Unit, Yarra Bend Road Fairfield, Victoria, 3078, Australia; h Institute for Health Transformation, Deakin University, Geelong, Victoria, 3022, Australia; i Melbourne Medical School, Faculty of Medicine, Dentistry and Health Sciences, University of Melbourne, Parkville, Victoria, 3010, Australia; j Department of Medical Education, Melbourne Medical School, University of Melbourne, Parkville, Victoria, 3010, Australia; k Melbourne School of Population and Global Health, University of Melbourne, Parkville, Victoria, 3010, Australia

**Keywords:** First Nations, Midwifery continuity, Culturally tailored care, Clinical outcomes

## Abstract

**Background:**

Perinatal morbidity and mortality are substantially higher for Aboriginal and Torres Strait Islander (hereafter called First Nations) mothers and babies compared with non-First Nations peoples. Women birth in systems designed and informed by Western values, and many report negative interactions with health professionals and a lack of cultural safety in the mainstream maternity system. To redress unacceptable health outcomes and system challenges, we implemented a culturally tailored caseload midwifery care programme called Baggarrook Yurrongi, at three tertiary maternity services in Melbourne, Australia. The model included continuity throughout pregnancy, labour, birth and the early postnatal period, recognition of culture as central to identity, and respecting and acknowledging the cultural background, beliefs and values of First Nations peoples. This paper describes maternal and infant health outcomes.

**Methods:**

This prospective non-randomised translational study used routinely collected clinical outcome data to explore whether, for women expecting a First Nations baby, receiving the new model was associated with improved clinical outcomes compared with usual care prior to and since implementation (adjusted for age, Body Mass Index, marital status, parity, diabetes and hypertensive disorders). Specifically, would the model decrease the proportion of First Nations babies born low birthweight (<2500 g) and increase the proportion born ‘healthy’ (alive, at term, of normal weight and size, and not admitted to neonatal special or intensive care (NICU)). All births were included except multiple pregnancies, where babies had major congenital anomalies, and births occurring within the first six months of model implementation.

**Findings:**

Baseline data were from 2012 to model commencement (2017) (99,952 non-First Nations and 1159 First Nations births). ‘After’ data were collected to 2022 (62,499 non-First Nations and 1038 First Nations births), and 669/1038 eligible women (64.5%) received the Baggarrook Yurrongi model. Fewer First Nations babies whose mothers received the model compared with those who received usual care ‘Before’ were low birthweight (AOR 0.67, 95% CI 0.47, 0.93) and more were born ‘healthy’ (AOR 1.45, 95% CI 1.14, 1.84).

**Interpretation:**

Culturally tailored caseload midwifery care significantly improved perinatal outcomes for women and their First Nations babies. Given poor perinatal outcomes are major risk factors affecting short- and long-term health, we recommend widespread model implementation, adapted to the needs of local First Nations communities, and supported by policymakers. Future research should include monitoring implementation and outcomes of the programme, along with robust cost-effectiveness analysis data to inform scale-up.

**Funding:**

Australian National Health and Medical Research Council.

**Research in context**

**Evidence before this study**

Perinatal mortality is substantially higher for Australian First Nations babies than non-First Nations babies. Preterm birth and low birthweight are key contributors to childhood disability and mortality and associated with preventable chronic diseases such as diabetes, renal and heart disease, all of which are overrepresented in the First Nations population. Given these health disparities, numerous government reports have called for strategies to improve outcomes for First Nations mothers and babies. Before this study, a Cochrane review including over 17,000 women reported that women who had midwife-led continuity of care (compared with standard maternity care) were less likely to experience preterm birth or fetal loss prior to 24 weeks’ gestation. Few studies in the review included women at high risk of complications, and none explicitly included women with social risk factors, with the review concluding that future research should focus on these groups. Since project commencement, we have had an ongoing search alert for relevant Medical Subject Headings (MeSH) search terms and actively monitored publications, therefore no additional specific literature search was conducted for this study.

**Added value of this study**

We found that culturally tailored continuity of midwifery care was associated with improved perinatal outcomes for women and their First Nations babies. These findings, considered alongside other key outcomes of the overarching study—reported high satisfaction and cultural safety among women, strong model uptake, and midwives’ positive experiences delivering care—underscore the value of this model. Our findings add to the evidence from previous studies that have explored the impact of continuity of care models for First Nations women, and in particular the large prospective study in Brisbane, Australia, that reported multiple improved outcomes for First Nations families through implementation of a multifaceted culturally tailored caseload midwifery model that reduced preterm birth and NICU admissions, and increased exclusive breastfeeding on discharge from hospital. Our before and after analysis of the gap in outcomes between women having First Nations and non-First Nations babies is the first reported in such a large study.

**Implications of all the available evidence**

Our findings support Australian government policies that recommend increased access to culturally tailored, accessible and affordable care in pregnancy as well as investment in workforce to support the models. Scale-up such as that reported here must be prioritised by Australian governments so that all women having a First Nations baby receive culturally tailored midwifery continuity models. It is possible the findings presented here may have similar outcomes for First Nations peoples (and potentially other priority population groups) in other countries, and we suggest models like this, tailored to local needs, be implemented within appropriate evaluation frameworks. Future research should include broad monitoring of the implementation of the programme (and barriers to scale-up), and site-specific evaluations that explore First Nations community views and experiences of the model, along with clinical outcomes and cost-effectiveness analyses.

## Introduction

Australia is one of the safest places in the world to give birth, however inequality exists, and Aboriginal and Torres Strait Islander (hereafter called First Nations) peoples experience poorer perinatal outcomes than non-First Nations individuals.^1^ There is an urgent need to improve health outcomes for First Nations mothers and babies in Australia. The enduring effects of colonisation and ongoing discrimination and institutional racism on health, wellbeing and the social determinants of health contribute to the inequity experienced by First Nations families.^2^ Structural factors also have an impact—circumstances such as housing instability, chronic stress, and reduced access to services contribute to poorer perinatal outcomes through the accumulation of stressors and systemic barriers to culturally safe care.^3^ Women birth in systems designed and informed by Western values that exclude Indigenous knowledges and practices.^2^ Negative interactions with health professionals and a lack of cultural safety (where an individual’s cultural identity and wellbeing is impacted^4^) in the mainstream maternity system have been reported by many First Nations women.^5^^,^^6^ Perinatal mortality is substantially higher for First Nations babies than non-First Nations babies (18.4 versus 10.5 deaths/1000 births) and maternal mortality three times higher (19.0 versus 5.5 deaths/100,000 births).^1^ Preterm birth (birth <37 completed gestational weeks) is almost double compared with non-First Nations infants (13.0% versus 8.4%) and likewise low birthweight (<2500 g (g); 13.0% versus 7.2%), and both are associated with significant morbidity and mortality.^1^ Preterm birth is a key contributor to childhood disability and mortality and associated with preventable chronic diseases such as diabetes, renal and heart disease,^7^ all of which are overrepresented in the First Nations population.^8^ Babies born preterm and of low birthweight are also at higher risk of adverse neurodevelopmental outcomes.^7^ Smoking in pregnancy (associated with preterm birth, stillbirth and sudden infant death syndrome^9^) is substantially higher (42.2% versus 10.9%), and smoking cessation in pregnancy lower (10.1% versus 26.9%) among First Nations women.^1^ Breastfeeding initiation is also lower (83% versus 91%).^1^ In Australia in 2023, First Nations mothers accounted for 5.6% (15,802) of women who gave birth, and First Nations babies (i.e., where either or both parents were First Nations) accounted for 7.2% (20,439) of all births.^1^

Given the health disparities, government reports call for strategies to improve outcomes.^8^^,^^10^ One of the national ‘Closing the Gap’ targets is to increase the proportion of babies born (and continuing) healthy and strong (i.e., reducing infant mortality and morbidity).^8^ One effective strategy to improve health outcomes for mothers and babies is midwifery continuity of care, where women receive continuity in pregnancy, labour, birth and postnatally from the same known midwife, although the most recent systematic review on midwife continuity reports that few studies have included women at high risk of complications, and none explicitly include women with social risk factors.^11^

Despite the benefits of midwifery continuity, and the substantial inequities experienced by many First Nations women and babies, few Australian women having a First Nations baby receive this model of care.^1^ One study in Queensland introduced a culturally tailored midwife continuity model (incorporating First Nations governance through a multiagency partnership, a First Nations workforce providing wraparound services and a community-based care hub) and found increased antenatal attendance and breastfeeding, lower medical interventions, and reduced preterm birth, low birthweight, and neonatal admissions.^12^

Our team evaluated the capacity of four maternity services in Victoria, Australia, to design, implement, embed, and sustain new culturally tailored caseload midwifery models of care for women having a First Nations baby.^13^ The study and overarching programme was called Baggarrook Yurrongi, meaning ‘Woman’s Journey’ in the local First Nations Woiwurrung language.^13^ We use the term ‘culturally tailored’ care to describe care that recognises culture as central to identity, and that respects and acknowledges the cultural background, beliefs and values of First Nations peoples. We do not use the terminology ‘culturally safe care’ because cultural safety (the experience of feeling safe, connected to culture and cultural identity) is determined by the First Nations individual themselves—if a service does not feel safe to the recipient, it is not culturally safe.^14^

The primary aim of the programme overall (described below) was to increase the proportion of women having a First Nations baby who were proactively offered and received caseload care. This was achieved at three major metropolitan tertiary maternity services in Melbourne, resulting in a 21-fold increase in access to caseload care (from 34 First Nations women ever receiving caseload midwifery care within the services to more than 700 over the three year study period).^13^ The Baggarrook Yurrongi model was not implemented in the regional setting due to ongoing staffing challenges.^13^ Where implementation was successful, model uptake was high (90%),^13^ and overall, women were very satisfied with their care, reporting feeling culturally, clinically and emotionally safe.^15^^,^^16^ There was also improved identification of First Nations mothers and babies,^17^ and high satisfaction among midwives (acknowledging the need for monitoring of caseload numbers and organisational support).^18^

This paper reports on a key secondary outcome of the Baggarrook Yurrongi study—the effect on maternal and infant health outcomes.

## Methods

### Study design

Baggarrook Yurrongi was a prospective non-randomised translational study in Victoria, Australia. In this paper, a before and after design is used to compare cross-sectional routinely collected de-identified clinical outcome data prior to (‘Before’), and following (‘After’) implementation of the new models.

The models were developed in partnership with First Nations health units at each service and the Victorian Aboriginal Community Controlled Health Organisation (VACCHO), the peak body representing Aboriginal Community Controlled Health Organisations (ACCHOs) across Victoria. Women were allocated a primary midwife and 1–2 back up midwives who provided pregnancy, labour, birth and early postnatal (one to two weeks postpartum) care. Women had 24-h access to their midwife, who was also on call for labour and birth. The caseload model was different to usual caseload models in that it was provided in conjunction with existing supports for First Nations women through community- and hospital-based First Nations health services, including the Victorian Koori Maternity Service,^19^ other ACCHO services, and hospital-based First Nations health workers, with whom the caseload midwives regularly liaised. Care was tailored at each site and for individual women, and provided in collaboration with obstetricians and other health professionals as required. Care could be solely hospital-based, with all care by the caseload midwife, or shared care between the caseload midwife and other specialised hospital service, e.g., maternal fetal medicine unit, obstetric team etc. Women could also choose a shared care option with their midwife and their local ACCHO. All women having a First Nations baby were eligible to be offered the model, regardless of risk factors. Each of the three study sites named their respective model based on local consultation. The Royal Women’s Hospital (the Women’s) was Baggarrook, Mercy Hospital for Women (the Mercy)—Nangnak, and Joan Kirner Women’s and Children’s Hospital (Joan Kirner)—Galinjera.

#### The model was designed to enhance, not replace, existing systems

All caseload midwives, managers, and some obstetric staff working within the model of care participated in in-house cultural training as well as cultural safety training in First Nations health conducted by VACCHO, including face-to-face and online. The aim was to assist staff understand the impact of colonial history on First Nations people and the context of women’s lives, and explore how to work effectively with First Nations people and their communities.^13^ The training included information on working with the Victorian First Nations community health sector, and on closing the health gap between First Nations and non-First Nations people. Women had access to informal and formal engagement with their respective site-based Aboriginal Health Units and staff, and anecdotally many took up this opportunity. Whenever possible the services employed First Nations midwives to provide the care, and likewise facilitated clinical placements for First Nations student midwives.

Prior to the model design and implementation, usual care varied by site and what was available at the site, but in general, for women having a First Nations baby, there was limited or no access to continuity of midwife care and 24-h access to a known midwife, and no systematic access to culturally tailored care.^13^ During the implementation period described here, most women who did not receive the culturally tailored caseload midwifery received standard hospital care, including specialist obstetric or perinatal care or standard midwife care, with a small number cared for in the drug and alcohol unit.^13^

### Ethics

The study was conducted according to the ethical principles in the Declaration of Helsinki. Research ethics approval was obtained from St Vincents Hospital (overall multisite approval, 16∖SVHM∖233) and La Trobe University (HREC 195/16), with site specific approval (SSA) from the three study sites: the Royal Women’s Hospital (SSA/17/RWHV/6), Mercy Hospital for Women (2017-028), and Western Health (for Joan Kirner Women’s and Children’s Hospital 16∖SVHM∖233∖SSA). The study was conducted in accordance with National Guidelines for Ethical Conduct in Aboriginal and Torres Strait Islander Research,^20^ and the team included a number of First Nations researchers, health workers and clinicians who were part of shaping the research questions and areas of study as well as in interpreting all study findings. For example, First Nations midwives led the exploration of women’s views^15^^,^^16^^,^^21^ and breastfeeding outcomes.^22^ The analyses presented in this paper were undertaken on anonymised routinely collected data, therefore the requirement for written informed consent from included individuals was waived.

### Participants

**Fig. 1:**
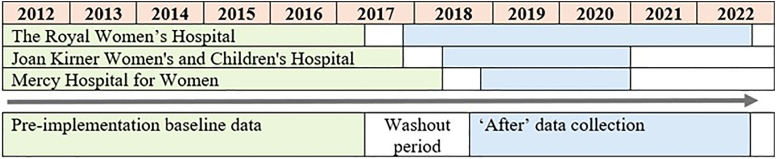
**Implementation and data collection timeline**.

Pre-implementation data were collected from 2012 up to commencement of the model at each site, then data were collected for all births after model commencement at the sites, continuing until the end of planned data collection (Fig. 1). We aimed to include the maternal and neonatal clinical outcome data of all women having a First Nations baby at the sites during the study period, excluding: a) births occurring within the first six months of model implementation (the ‘washout’ period), b) babies with major congenital anomalies, and c) multiple pregnancies. We collected the same data for all non-First Nations women having a non-First Nations baby to enable secondary analyses comparing the groups. Given the required sample size (details below), we extended data collection at the largest site until August 2022 to maximise available data and increase the precision of estimates of association.

### Outcomes and procedures

#### Primary aims and outcomes

Our primary aim was to determine whether, for women expecting a First Nations baby, receiving the new culturally tailored model was associated with improved maternal and neonatal outcomes compared with receiving usual care prior to implementation. Specifically, would the model: decrease the proportion of First Nations infants born low birthweight (<2500 g) (primary clinical outcome), small for gestational age (<10th centile), preterm (<37 weeks), admitted to neonatal special or intensive care unit (NICU) or stillborn; increase breastfeeding initiation; and decrease the proportion of women having a First Nations baby who smoked tobacco >20 weeks gestation. We also aimed to determine if the new model would increase the proportion of First Nations infants born ‘healthy’ (alive, at term, of normal weight and size, and not admitted to NICU)—a strengths-based outcome. A range of other routine clinical outcomes were explored: type of birth, obstetric analgesia, labour onset, postpartum haemorrhage, perineal trauma, length of hospital stay, and other measures of neonatal and maternal morbidity and mortality.

#### Secondary aims and outcomes

As pre-specified prior to study commencement we explored the same outcomes using different comparison groups.

1. Any disparities in outcomes between First Nations mothers and babies and non-First Nations mothers and babies at baseline ‘Before’ implementation were compared with any disparities in outcomes ‘After’ implementation (for babies whose mother received caseload care), to explore any changes over time.
2. First Nations mothers and babies who received the caseload care ‘After’ implementation were compared with those who did not receive the new model in that period, to account for potential confounding related to system-wide changes over time.
3. The outcomes of *all* First Nations mothers and babies ‘Before’ implementation were compared with *all* First Nations mothers and babies ‘After’ implementation, regardless of model of care received.

We also undertook sensitivity analyses examining the same outcomes but including only First Nations babies whose mothers also identified as First Nations, excluding those with non-First Nations mothers. This approach accounts for the possibility that changes over time in how First Nations status is defined (shifting from maternal identification to the baby’s status) could artificially suggest improvements in outcomes without real change,^23^ potentially undermining efforts to achieve equity.^24^ Additionally, although mostly similar, some outcomes (e.g., low birthweight, preterm birth) may be slightly less prevalent among First Nations babies with non-First Nations mothers compared to those with First Nations mothers, potentially reflecting a difference in perinatal risk factors^23^ of which care providers should be aware. We explored this to provide a comprehensive picture of the potential effects of culturally tailored caseload midwifery models.

#### Sample size

Sample size calculations were undertaken assuming ≈200 women having a First Nations baby were birthing at the combined sites each year. Allowing time for the model to be designed, implemented and embedded (we collected, but *do not report on*, outcome data for the first six months of model implementation at each site), we aimed to obtain post-implementation data for up to 650 First Nations babies whose mother had received the new model. We planned to obtain the same data for at least the previous 650 First Nations babies born at the sites *prior to study commencement* to use as a baseline. Before the study 13.3% of First Nations babies at the combined sites were low birthweight (compared with 7.1% of non-First Nations babies). Data for 650 babies ‘Before’ and ‘After’ allowed detection of a decrease of 5% in the rate of low birthweight down to 8.3% (80% power, 95% confidence). We chose the proportion of First Nations babies born a healthy birthweight as the primary outcome for the sample size calculation in line with Target 2 of the national Closing the Gap strategy—that 91% of First Nations babies be born a healthy birthweight.^25^ These numbers also allowed detection of a decrease in preterm birth (14.4%–8.2%), NICU admission (21.1%–15.0%) and smoking >20 weeks (26.5%–19.3%). The sample size was insufficient to determine statistical differences in rare morbidities and mortality.

#### Statistical analysis

De-identified routinely collected electronic data were provided from the three services in Excel spreadsheets then downloaded to STATA 18 for cleaning and analysis. Most of the variables provided are part of the mandatory data collected by the State Government for all births in Victoria, and data quality checks are undertaken at multiple points by both health services and the government perinatal data unit. Quantitative data were summarised using frequencies and percentages, and groups compared using odds ratios (ORs), with 95% confidence intervals (CIs) to describe associations (ORs were chosen to facilitate ready comparison with similar articles in the field). Small for gestational age was derived using Australian birthweight centiles.^26^ We adjusted for covariates available in the routinely collected data that potentially affected the various outcome variables, including maternal age, parity (first baby or not), body mass index (BMI), and diabetes and/or hypertension in pregnancy (and provide adjusted ORs, AORs). The proportion of missing data was small, with less than 1% missing for all characteristics and outcome variables, except initiation of breastfeeding (1.9%) and BMI (4.3%), both of which had less than 5% missing data. In the regression analysis a complete case analysis was conducted, that is, only participants with complete data across all covariates were included. No multiplicity adjustment was conducted, therefore the results are exploratory, and confidence intervals are unadjusted. All data analyses conducted were pre-specified. Primary and secondary analyses included all First Nations babies even where their mother was non-First Nations. *Sensitivity analyses* explored the impact on outcomes if including only women who were First Nations.^23^ Improved identification of First Nations status during the project^17^ meant that the percentage of non-First Nations mothers of First Nations babies increased from 14%, 17% and 9% at the sites in the ‘Before’ cohorts to 29%, 36% and 25% in the ‘After’ cohorts. It could therefore potentially be that any improved outcomes were, in part, due to increased numbers of non-First Nations mothers, as reported elsewhere.^23^^,^^24^

#### Role of the funding source

The funder of the study had no role in study design, data collection, data analysis, data interpretation, or writing of the report.

## Results

Baseline (‘Before’) data were obtained for the period from the start of 2012 until commencement of the model at the respective sites (Fig. 1). At the Women’s, the model commenced in March 2017, with post-implementation (‘After’) data analysed for births occurring from September 2017 to August 2022 inclusive. Joan Kirner commenced in October 2017, with ‘After’ data analysed from births from April 2018 to December 2020 inclusive. The Mercy commenced mid-April 2018, with ‘After’ data analysed from births from mid-October 2018 to December 2020 inclusive.

In total, 164,648 women and their babies are included in the various analyses. Not including the six-month washout period, 1531 women and their baby were excluded due to the baby having a severe congenital anomaly (46 First Nations babies and 1485 non-First Nations babies) and 3431 women had a multiple birth (50 women having a First Nations baby and 3381 having a non-First Nations baby). Total available births in the ‘Before’ data were 99,952 non-First Nations and 1159 First Nations births and in the ‘After’ data, 62,499 non-First Nations and 1038 First Nations births. Table 1 shows the characteristics of all women included in the analysis, with women in the ‘After’ period divided by those who received the caseload model (n = 669/1038, 64.5%) versus those that did not (n = 369/1038, 35.5%).

**Table 1:** Characteristics and birth outcomes of mothers of First Nations babies compared to mothers of non-First Nations babies, with ‘After’ data separated by receiving the caseload model or not for women having a First Nations baby.

Characteristic/outcome	‘Before’		‘After’		
	First Nations baby		First Nations baby		
	No n (%) n = 99,952	Yes n (%) n = 1159	No n (%) n = 62,499	Yes	
				Received caseload	
				No n = 369, n (%)	Yes n = 669, n (%)
Age (yrs—mean, sd)	n = 99,946, 31.5 (5.1)	27.3 (6.1)	n = 62,499, 32.4 (4.9)	n = 369, 29.2 (5.9)	n = 669, 28.3 (5.7)
Partnered/married	n = 99,334, 87,986 (88.6)	n = 1143, 662 (57.9)	n = 62,190, 55,765 (89.7)	n = 364, 228 (62.6)	n = 666, 415 (62.3)
Primiparous	n = 99,952, 47,302 (47.3)	n = 1159, 471 (40.6)	n = 62,499, 30,673 (49.1)	n = 369, 130 (35.2)	n = 669, 271 (40.5)
Smoking < 20 wks.	n = 99,645, 5592 (5.6)	n = 1144, 432 (37.8)	n = 62,412, 2437 (3.9)	n = 364, 119 (32.7)	n = 664, 192 (28.9)
Body mass index	n = 95,994	n = 1067	n = 59,656	n = 311	n = 617
<18.5	3008 (3.1)	32 (3.0)	1574 (2.6)	12 (3.9)	21 (3.4)
18.5–24.9	50,536 (52.6)	450 (42.2)	30,528 (51.2)	104 (33.4)	227 (36.8)
25–35	34,906 (36.4)	418 (39.2)	22,622 (37.9)	122 (39.2)	233 (37.8)
>35	7544 (7.9)	167 (15.7)	4932 (8.3)	73 (23.5)	136 (22.0)
Diabetes in pregnancy^a^	n = 99,952, 11,878 (11.9)	n = 1159, 135 (11.7)	n = 62,499, 9549 (15.3)	n = 369, 46 (12.5)	n = 669, 91 (13.6)
Hypertensive disorder in pregnancy	n = 99,952, 5877 (5.9)	n = 1159, 87 (7.5)	n = 62,499, 2991 (4.8)	n = 369, 22 (6.0)	n = 669, 32 (4.8)
Birth outcomes					
Gestation in weeks *(mean, sd)*	n = 99,950, 38.8 (2.4)	n = 1159, 37.9 (3.3)	n = 62,498, 38.6 (2.4)	n = 369, 37.1 (3.9)	n = 669, 38.3 (2.3)
Type of birth	n = 99,949	n = 1159	n = 62,499	n = 368	n = 669
Spontaneous vaginal	54,145 (54.2)	690 (59.5)	29,862 (47.8)	198 (53.8)	385 (57.6)
Instrumental vaginal	15,088 (15.1)	122 (10.5)	10,667 (17.1)	40 (10.9)	71 (10.6)
Caesarean section	30,716 (30.7)	347 (29.9)	21,970 (35.2)	130 (35.3)	213 (31.8)
Induction or augmentation^b^	n = 99,952, 44,043 (44.1)	n = 1159, 537 (46.3)	n = 62,499, 30,156 (48.3)	n = 369, 167 (45.3)	n = 669, 350 (52.3)
Epidural analgesia^c^	n = 99,952, 25,366 (25.4)	n = 1159, 308 (26.6)	n = 62,499, 20,246 (32.4)	n = 369, 100 (27.1)	n = 669, 180 (26.9)
Third or fourth degree tear^d^	n = 69,236, 2573 (3.7)	n = 812, 12 (1.5)	n = 40,529, 1698 (4.2)	n = 239, 6 (2.5)	n = 456, 13 (2.9)
Postpartum haemorrhage n = 99,910		n = 1157	n = 62,487	n = 369	n = 669
EBL^e^ ≥ 1500	2292 (2.3)	24 (2.1)	1875 (3.0)	12 (3.3)	16 (2.4)
EBL ≥ 1000	6173 (6.2)	74 (6.4)	5291 (8.5)	39 (10.6)	47 (7.0)

NB: denominators not provided as differing missing data across variables, but % reflect correct denominators for all outcomes.

a Any diabetes.

b Induction and augmentation data unable to be separated for all sites.

c Includes all births as not possible to reliably ascertain onset of labour and omit women with no labour.

d Vaginal births only.

e EBL—estimated blood loss.

Table 2 shows the primary analysis. There were fewer babies born low birthweight (AOR 0.67, 95% CI 0.47, 0.93), preterm (AOR 0.64, 95% CI 0.47, 0.87) or admitted to NICU (AOR 0.70, 95% CI 0.55, 0.90), more babies born ‘healthy’ (AOR 1.45, 95% CI 1.14, 1.84), and more women who initiated breastfeeding (AOR 1.86, 95% CI 1.29, 2.67) compared with pre-implementation.

**Table 2:** Primary analysis—key clinical outcomes where mothers of First Nations babies received caseload care (‘After’ period) compared to ‘Before’ period when model not in place.

Outcome	First Nations baby Before (usual care)	First Nations baby After (had caseload)	OR (CI)	Adj^d^ OR (CI)
	n (%), n = 1159	n (%), n = 669		
Low birthweight				
Yes	182 (15.7)	70 (10.5)	0.63 (0.47, 0.84)	0.67 (0.47, 0.93)
No	976 (84.3)	599 (89.5)	Ref	Ref
SGA				
Yes	23 (2.0)	12 (1.8)	0.90 (0.45, 1.82)	0.89 (0.43, 1.86)
No	1136 (98.0)	657 (98.2)	Ref	Ref
Preterm birth				
Yes	210 (18.1)	86 (12.9)	0.67 (0.51, 0.87)	0.64 (0.47, 0.87)
No	949 (81.9)	583 (87.1)	Ref	Ref
Admitted NICU^a^				
Yes	328 (28.7)	145 (21.7)	0.69 (0.55, 0.87)	0.70 (0.55, 0.90)
No	817 (71.4)	522 (78.3)	Ref	Ref
Stillborn				
Yes	13 (1.1)	2 (0.3)	0.26 (0.06, 1.17)	0.26 (0.03, 2.20)
No	1146 (98.9)	667 (99.7)	Ref	Ref
Healthy infant^b^				
Yes	774 (66.8)	500 (74.7)	1.47 (1.19, 1.82)	1.45 (1.14, 1.84)
No	385 (33.2)	169 (25.3)	Ref	Ref
Smoking >20 weeks				
Yes	343 (30.9)	175 (26.8)	0.82 (0.66, 1.01)	0.84 (0.66, 1.06)
No	766 (69.1)	478 (73.2)	Ref	Ref
BF^c^ initiation				
Yes	961 (85.9)	597 (90.7)	1.61 (1.18, 2.20)	1.86 (1.29, 2.67)
No	158 (14.1)	61 (9.3)	Ref	Ref

SGA—small for gestational age.

a Neonatal special or intensive care (NICU, data not able to be separated).

b Born alive, at term, of normal weight and size, and not admitted to NICU.

c Breastfeeding.

d Adjusted for age (continuous), Body Mass Index (BMI, group), marital status, parity, diabetes and hypertensive disorder. NB: denominators differ slightly across variables, but % reflect correct denominators for all outcomes.

Table 3 shows outcomes for First Nations mothers and babies compared with non-First Nations mothers and babies at baseline (‘Before’), looking at the disparities between the groups, then First Nations mothers and babies (where the woman had caseload care) compared with non-First Nations mothers and babies ‘After’, looking at the disparities between the groups. Supplementary Table S1 (S1) provides demographic characteristics of the women in both groups. Across all the outcomes except small for gestational age, AORs and 95% CIs indicate an improvement in the ‘After’ period.

**Table 3:** Key clinical outcomes of First Nations mothers and babies compared with non-First Nations mothers and babies, showing ‘Before’ and ‘After’ outcomes (where mothers of First Nations babies received usual care ‘Before’ and tailored caseload care ‘After’).

Outcome	Before				After			
	First Nations baby		OR (CI)	Adj^d^ OR (CI)	First Nations baby		OR (CI)	Adj^d^ OR (CI)
	No, n (%)	Yes, n (%)			No, n (%)	Yes, n (%)		
Low birthweight								
Yes	7144 (7.2)	182 (15.7)	2.42 (2.06, 2.84)	2.04 (1.70, 2.45)	4425 (7.1)	70 (10.5)	1.53 (1.20, 1.97)	1.30 (0.98, 1.74)
No	92,765 (92.9)	976 (84.3)		Ref	58,069 (92.9)	599 (89.5)		Ref
SGA								
Yes	3477 (3.5)	23 (2.0)	0.56 (0.37, 0.85)	0.58 (0.37, 0.89)	1539 (2.5)	12 (1.8)	0.72 (0.41, 1.28)	0.85 (0.47, 1.51)
No	96,475 (96.5)	1136 (98.0)			60,960 (97.5)	657 (98.2)		
Preterm birth								
Yes	8098 (8.1)	210 (18.1)	2.51 (2.16, 2.92)	2.17 (1.83, 2.58)	4865 (7.8)	86 (12.9)	1.75 (1.39, 2.20)	1.45 (1.12, 1.89)
No	91,821 (91.9)	949 (81.9)		Ref	57,618 (92.2)	583 (87.1)		Ref
Admission to NICU^a^								
Yes	12,995 (13.1)	328 (28.7)	2.67 (2.34, 3.04)	2.26 (1.96, 2.61)	8564 (13.8)	145 (21.7)	1.73 (1.44, 2.08)	1.36 (1.10, 1.67)
No	86,378 (86.9)	817 (71.4)		Ref	53,351 (86.2)	522 (78.3)		Ref
Stillborn								
Yes	616 (0.6)	13 (1.1)	1.83 (1.05, 3.18)	1.23 (0.58, 2.61)	600 (1.0)	2 (0.3)	0.31 (0.08, 1.24)	0.17 (0.02, 1.23)
No	99,333 (99.4)	1146 (98.9)		Ref	61,891 (99.0)	667 (99.7)		Ref
Healthy infant^b^								
Yes	83,126 (83.2)	774 (66.8)	0.41 (0.36, 0.46)	0.49 (0.43, 0.57)	51,496 (82.4)	500 (74.7)	0.63 (0.53, 0.75)	0.81 (0.66, 0.98)
No	16,826 (16.8)	385 (33.2)		Ref	11,003 (17.6)	169 (25.3)		Ref
Smoking >20 weeks								
Yes	4296 (4.4)	343 (30.9)	9.85 (8.64, 11.23)	4.73 (4.06, 5.52)	2090 (3.4)	175 (26.8)	10.52 (8.80, 12.58)	4.64 (3.76, 5.74)
No	94,529 (95.7)	766 (69.1)		Ref	60,059 (96.6)	478 (73.2)		Ref
BF^c^ initiation								
Yes	94,325 (95.9)	961 (85.9)	0.26 (0.22, 0.31)	0.48 (0.39, 0.58)	58,797 (96.1)	597 (90.7)	0.40 (0.31, 0.52)	0.86 (0.62, 1.18)
No	3999 (4.1)	158 (14.1)		Ref	2398 (3.9)	61 (9.3)		Ref

SGA—small for gestational age.

a Neonatal special or intensive care (NICU, data not able to be separated).

b Born alive, at term, of normal weight and size, and not admitted to NICU.

c BF—breastfeeding.

d Adjusted for age (continuous), BMI (group), marital status, parity, diabetes and hypertensive disorder. NB: denominators differ slightly across variables, but % reflect correct denominators for all outcomes.

**Fig. 2:**
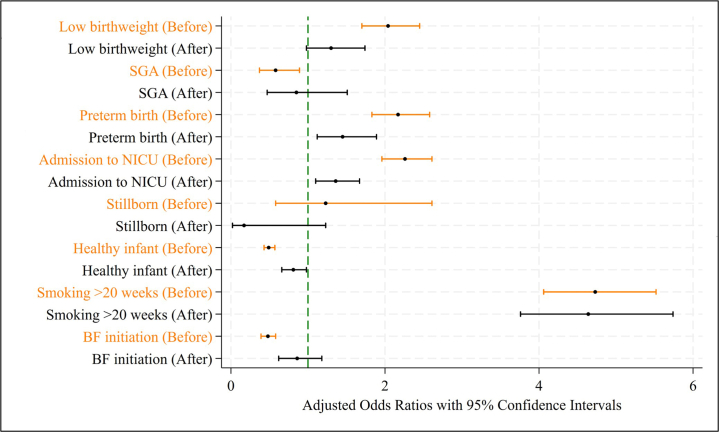
**Graphic representation of comparison of ‘Before’ and ‘After’ AORs of key clinical outcomes of First Nations mothers and babies compared with non-First Nations mothers and babies, (where mothers of First Nations babies received usual care ‘Before’ and tailored caseload care ‘After’).** AOR—adjusted odds ratio. SGA—small for gestational age. NICU—Neonatal special or intensive care. BF–breastfeeding.

Fig. 2 shows the same data graphically.

Table 4 shows a comparison of clinical outcomes in the ‘After’ period only—First Nations mothers and babies who received the Baggarrook Yurrongi model compared with those who did not (to account for potential confounding associated with system changes over time). There were improved outcomes across all measures where women received the model, except babies being born small for gestational age and mothers smoking after 20 weeks gestation. Demographic characteristics of mothers who received the model (versus not) were very similar (Table 1), and there was no systematic bias in who accessed the model. A small number of women transferred to another hospital before being offered the model, a small number were not identified as First Nations until after birth and some women were inadvertently not offered the model by staff. The main reason women were unable to access the model across all sites a lack of midwife availability, due to planned or unplanned leave or to staff turnover, in combination with increased numbers of First Nations women seeking out the three services in order to access the model. Most of these women received standard hospital care including specialist obstetric or perinatal care, standard midwife care or in a specialised clinic.^13^

**Table 4:** Clinical outcomes of First Nations mothers and babies in the ‘After’ period, comparing women who received caseload care with those who did not.

Outcome	Received caseload No, n (%), (n = 369)	Received caseload Yes n (%), (n = 669)	OR (CI)	Adj. OR^d^ (CI)
Low birthweight				
Yes	72 (19.5)	70 (10.5)	0.48 (0.34, 0.69)	0.47 (0.31, 0.71)
No	297 (80.5)	599 (89.5)	Ref	Ref
SGA				
Yes	6 (1.6)	12 (1.8)	1.11 (0.41, 2.97)	1.50 (0.47, 4.81)
No	363 (98.4)	657 (98.2)	Ref	Ref
Preterm birth *(n = 368/669)*				
Yes	74 (20.1)	86 (12.9)	0.59 (0.42, 0.82)	0.57 (0.39, 0.86)
No	294 (79.9)	583 (87.1)	Ref	Ref
Admitted to NICU^a^*(n = 357/667)*				
Yes	126 (35.3)	145 (21.7)	0.51 (0.38, 0.68)	0.51 (0.37, 0.71)
No	231 (64.7)	522 (78.3)	Ref	Ref
Stillborn				
Yes	12 (3.3)	2 (0.3)	0.09 (0.02, 0.40)	0.07 (0.01, 0.58)
No	357 (96.8)	667 (99.7)	Ref	Ref
Healthy infant^b^				
Yes	216 (58.5)	500 (74.7)	2.10 (1.60, 2.75)	2.04 (1.50, 2.78)
No	153 (41.5)	169 (25.3)	Ref	Ref
Smoking >20 weeks *(n = 357/653)*				
Yes	108 (30.3)	175 (26.8)	0.84 (0.64, 1.12)	0.85 (0.61, 1.18)
No	249 (69.8)	478 (73.2)	Ref	Ref
BF^c^ initiation *(n = 350/658)*				
Yes	293 (83.7)	597 (90.7)	1.90 (1.29, 2.80)	2.18 (1.37, 3.47)
No	57 (16.3)	61 (9.3)	Ref	Ref

SGA—small for gestational age.

a Neonatal special or intensive care (NICU, data not able to be separated).

b Born alive, at term, of normal weight and size, and not admitted to NICU.

c BF—breastfeeding.

d Adjusted for age (continuous), BMI (group), marital status, parity, diabetes and hypertensive disorder.

Table 5, the final secondary analysis table, shows the same outcomes as Table 2, but here we compare data at baseline, that is the outcomes of First Nations mothers and babies ‘Before’ model implementation, with all First Nations mothers and babies in the ‘After’ period regardless of whether the woman received the Baggarrook Yurrongi model (369/1038, 35.5% did not). There was a proportional improvement across most outcomes (as in Table 2), but the only strong association was improved breastfeeding initiation.

**Table 5:** Key clinical outcomes for First Nations mothers and babies ‘Before’ and ‘After’ (regardless of whether or not caseload model received in ‘After’ period).

Outcome	First Nations baby Before n (%), (n = 1159)	First Nations baby After n (%), (n = 1038)	OR (95% CI)	Adj^d^ OR (95% CI)
Low birthweight *(n = 1158/1038)*				
Yes	182 (15.7)	142 (13.7)	0.85 (0.67, 1.08)	0.89 (0.68, 1.18)
No	976 (84.3)	896 (86.3)		Ref
SGA				
Yes	23 (2.0)	18 (1.7)	0.87 (0.47, 1.62)	0.79 (0.40, 1.55)
No	1136 (98.0)	1020 (98.3)		
Preterm birth *(n = 1159/1037)*				
Yes	210 (18.1)	160 (15.4)	0.82 (0.66, 1.03)	0.79 (0.61, 1.03)
No	949 (81.9)	877 (84.6)		Ref
Admitted to NICU^a^				
Yes	328 (28.7)	271 (26.5)	0.90 (0.74, 1.08)	0.89 (0.72, 1.09)
No	817 (71.4)	753 (73.5)		Ref
Stillborn				
Yes	13 (1.1)	14 (1.4)	1.21 (0.56, 2.58)	1.56 (0.55, 4.49)
No	1146 (98.9)	1024 (98.7)		Ref
Healthy infant^b^				
Yes	774 (66.8)	716 (69.0)	1.11 (0.92, 1.32)	1.13 (0.92, 1.38)
No	385 (33.2)	322 (31.0)		Ref
Smoking >20 weeks *(n = 1109/1010)*				
Yes	343 (30.9)	283 (28.0)	0.87 (0.72, 1.05)	0.89 (0.72, 1.10)
No	766 (69.1)	727 (72.0)		Ref
BF^c^ initiation *(n = 1119/1008)*				
Yes	961 (85.9)	890 (88.3)	1.24 (0.96, 1.60)	1.39 (1.03, 1.87)
No	158 (14.1)	118 (11.7)		Ref

SGA—small for gestational age.

a Neonatal special or intensive care (NICU, data not able to be separated).

b Born alive, at term, of normal weight and size, and not admitted to NICU.

c BF—breastfeeding.

d Adjusted for age (continuous), BMI (group), marital status, parity, diabetes and hypertensive disorder.

Sensitivity analyses explored whether outcomes were different for First Nations babies whose mothers were themselves First Nations compared with those who were not. Table 6 shows the number and proportion of First Nations mothers and babies in the ‘Before’ and ‘After’ data, demonstrating that after model implementation (and the concurrent focus on identification throughout this period), more First Nations babies were identified whose mother was non-First Nations (1159/101,111; 1.2% versus 1038/63,537; 1.6%). We compared the demographic characteristics of First Nations women having a First Nations baby with the non-First Nations women having a First Nations baby, along with the characteristics of the non-First Nations women having a non-First Nations baby (S1). A lower proportion of First Nations mothers of First Nations babies were primiparous (compared with non-First Nations mothers having a First Nations baby), and a higher proportion smoked before 20 weeks (in ‘Before’ and ‘After’ periods).

**Table 6:** Number and proportion of First Nations babies and mothers in ‘Before’ and ‘After’ data.

Mother/baby identification	Before		After	
	n	%	n	%
Mother First Nations	n = 101,213		n = 63,538	
	999	1.0	739	1.2
Baby First Nations	n = 101,111		n = 63,537	
	1159	1.2	1038	1.6

NB: In the ‘Before’ data there were more instances where baby status was not completed in the data. Babies with severe congenital anomalies and multiples are excluded from this table and the analyses.

Including only First Nations dyads (where both mother and baby were First Nations) in the same analysis shown in Table 2 showed proportional improvements in all outcomes, with strong evidence of improvement in preterm birth, the proportion of healthy infants and breastfeeding initiation (Supplementary Table S2). We were underpowered to show associations for the other outcomes given the much smaller sample size. Likewise, we analysed the outcomes as shown in Table 3, looking at the disparities between First Nations and non-First Nations outcomes ‘Before’ and ‘After’, but including only those First Nations dyads, and found evidence of improvement across all measures except (in this case) small for gestational age and smoking after 20 weeks (Supplementary Table S3). We also replicated the analysis shown in Table 4 included only First Nations dyads—comparing those who did and did not receive the new model in the ‘After’ period. Only small for gestational age was not improved (Supplementary Table S4). Finally, we did the same with the analysis shown in Table 5, comparing outcomes of First Nations dyads before model implementation, with all the First Nations dyads in the ‘After’ period regardless of whether the woman received the Baggarrook Yurrongi model (253/739, 34.2% did not). Here only breastfeeding initiation improved (Supplementary Table S5).

## Discussion

We found strong evidence of improved clinical outcomes for First Nations babies whose mothers received the Baggarrook Yurrongi culturally tailored model. We found higher odds of babies being born healthy (born alive, at term, normal weight and size, not admitted to NICU), with lower odds of preterm birth, low birthweight and admission to NICU, and increased breastfeeding initiation. Preterm birth and low birthweight are well established risk factors for short and long-term adverse consequences for infants, impacting on families and broader society.^7^ A 2023 *Lancet* series emphasised that preventing preterm birth and low birthweight could significantly advance global health and contribute to economic and social development, yet despite widespread recognition and international commitments in the past 30 years, progress in prevention has been limited.^7^ Given this, and limited implementation of effective strategies, alongside persistent high rates of preterm birth and low birthweight babies for First Nations peoples, our findings are particularly noteworthy. Likewise, our findings on increased breastfeeding initiation are important. Breastfeeding has short and long-term health benefits for both women and infants and has the largest potential impact on child mortality in comparison to any other preventative interventions.^27^ Breast milk is protective against many chronic diseases) that are major contributors to the mortality gap between First Nations and non-First Nations Australians.^8^

Our findings add to evidence from previous studies of the benefits of continuity of care models for First Nations women. A large prospective study implementing a multifaceted culturally tailored caseload midwifery model in Brisbane, Australia reported reduced preterm birth and NICU admissions, and increased exclusive breastfeeding on discharge from hospital.^28^ The Baggarrook Yurrongi outcomes were similar, and each of the three services had an Aboriginal Health Unit involved in model implementation that provided wrap-around services, and women had access to local ACCHOs, including the option of shared care as part of the model. Improved breastfeeding has also been reported in other studies implementing culturally tailored continuity of care for women having a First Nations baby.^29^^,^^30^ In the Baggarrook Yurrongi study, across the three sites, 18%, 50% and 53% respectively of women having a First Nations baby were at increased medical or obstetric risk,^31^ suggesting that not only is continuity of midwifery care safe for women at higher medical risk, but it is also protective. Midwifery continuity needs to be a strong element of any strategy to increase equity for First Nations families, yet the caseload model has not been available to most First Nations women, particularly where such models have stringent entry criteria related to perceived risk.

The Baggarrook Yurrongi model comprised multiple elements,^13^ and it is not possible to determine which specific element impacted the clinical outcomes. However, the model enhanced care coordination and communication, and was in clear contrast to fragmented mainstream care, where women usually receive care from multiple unfamiliar care providers and are often exposed to racism.^5^^,^^29^ Women who received the Baggarrook Yurrongi model were highly satisfied, and reported feeling more culturally and clinically safe, valuing the personalised care provided by a known midwife in partnership with First Nations staff within the respective Aboriginal Health Units.^16^ The Australian National Aboriginal and Torres Strait Islander Health Plan emphasises the importance of having a First Nations and/or culturally capable workforce given the centrality of culture on health outcomes,^10^ and in this study, midwives (and other key hospital staff) received cultural training. Caseload midwives were on-call to provide care during labour and birth and were available for women to call or text any time for advice or queries. Midwives knew women’s medical and social history, enabling earlier identification of issues, and implementation of additional care and collaboration as needed. This is similar to other studies of caseload midwifery where women reported being more likely to accept additional support when needed, such as mental health, drug/alcohol and social work support, and feeling more comfortable to talk to their midwives about general health issues such as nutrition, smoking and breastfeeding.^32^ For women experiencing complex social disadvantage (e.g., past trauma, social services involvement), a trusting relationship with a health care provider seems to be a key enabler for engaging with/accessing health care.^32^

The relationship between continuity of care, cultural safety and improved outcomes might be understood through several possible pathways. Continuity with a known midwife enables the development of trust, effective communication and advocacy, and more responsive, individualised care.^11^^,^^33^ When delivered within a culturally safe framework, this continuity may also reduce experiences of racism and power imbalance, while strengthening women’s sense of respect, identity and control.^34^ The improved perinatal outcomes we have seen in this study are plausibly mediated through increased engagement with care, earlier identification of risk, and more timely and appropriate intervention. Together, these mechanisms provide a coherent theoretical explanation for how culturally safe continuity models can improve outcomes, with potential applicability across diverse settings and populations.

We have previously published what we believe were key contributors to successful model implementation i.e., the model was First Nations’ community-driven and endorsed, with significant input and commitment from partners^13^ and hospital Aboriginal Health Units. There was First Nations leadership on the investigator team, and an Aboriginal Advisory Committee providing cultural guidance and promoting community engagement. Engagement with VACCHO and its member ACCHOs (e.g., Victorian Aboriginal Health Service and Koori Maternity Services) enabled shared care with the ACCHO sector, facilitating effective integration of hospital- and community-based services, and decreased the chance of women ‘falling through the cracks’. These key factors were also reported in the study by Kildea et al.^12^ This partnership approach is advocated by both government and the First Nations community-controlled sector and is fundamental in any strategy for improving health outcomes for First Nations peoples.

Our findings support Australian government policies that recommend strategies to improve health outcomes for First Nations mothers and babies. The National Aboriginal and Torres Strait Islander Health Plan (2021–2031) highlights the importance of culturally safe, accessible and affordable care in pregnancy to improve perinatal health, which requires health service redesign to meet community needs, and investment in workforce to support culturally safe models of care.^10^ The National Strategic Directions for Australian Maternity Services report also emphasises the need for services to provide appropriately developed, culturally safe and accessible perinatal care for First Nations women.^35^ Culturally tailored caseload midwifery models such as Baggarrook Yurrongi, developed in partnership with and by local First Nations communities, are an important strategy to address these policies. The national ‘Closing the Gap’ Implementation Plan 2025 has a target to increase the proportion of Aboriginal and Torres Strait Islander babies born with a healthy birthweight to 91% by 2031, and states that culturally safe maternity services are critical to this, and to maintaining the gains made toward improving the proportion of First Nations babies born healthy and strong.^8^ Scale-up of models such as that reported here must be prioritised by Australian governments so that all women having a First Nations baby receive culturally tailored midwifery continuity models. However, implementation at scale may present practical challenges. Continuity models require a stable and appropriately supported workforce, which may be difficult to achieve in some settings, particularly rural and remote areas, and sustaining culturally safe care also depends on long-term, secure funding and organisational commitment to model fidelity. Models should be developed in partnership with First Nations people and communities and use a framework that allows for customisation of the model to the local community context through a participatory action process, enabled by strong partnerships, and leadership from First Nations people and ACCHOs. This study was initiated by a First Nations organisation, with First Nations governance and research team members, and strong buy-in from all the Aboriginal Health Units at the three services. One aspect likely to improve outcomes further is formal partnerships with First Nations organisations, promoting better collaboration and data sharing and ongoing First Nations governance and community control. More also needs to be invested and tested to see what will lead to success in rural and remote areas where there are less resources and more disparity and inequity.^36^

A key strength of this study was that it was instigated in response to a request from VACCHO, who considered that First Nations women should have access to caseload midwifery care given the known beneficial clinical and psychosocial outcomes.^13^ Our First Nations partner organisations and Advisory Committee, along with the research team did not support implementing the model using a randomised controlled trial, therefore this study used a prospective translational before and after design. It is possible that the characteristics of women ‘Before’ and ‘After’ may have differed, and/or the women in the ‘After’ period who received caseload may have differed from those who did not. Additionally, the observed improvements may in part reflect secular trends or broader system-level changes over time, although we undertook pre-specified secondary analyses using contemporaneous comparison groups to mitigate this. We also adjusted for available key clinical covariates with the potential to affect outcomes, but other socioeconomic and structural factors not captured in routinely collected data may have had an effect. However, this was a large, carefully collected dataset with a focus on First Nations identification, and selection into the model was random. The multiple comparisons also had consistent findings—all showed evidence of improved outcomes. The only exception was in small for gestational age (SGA) outcomes, however a number of studies have suggested that SGA has a number of limitations as an accurate measure of infants at-risk.^37^ A 2025 multi-country study group undertook extensive analyses using varied methods and concluded that SGA—no matter how it is defined—is extremely limited as a category of defining risk and that the limitations should be acknowledged when interpreting SGA outcomes.^37^ Finally, this study was exploratory; multiple outcomes were examined without formal adjustment for multiplicity, so the findings should be interpreted with appropriate caution.

Our sensitivity analyses explored if we were under- or over-estimating effects given the increased percentage of First Nations babies with non-First Nations mothers in the ‘After’ period. These showed all outcomes in the same direction, with improved equity across all comparisons, and in particular, evidence of a very strong association with improved outcomes for First Nations dyads who received the new model compared with those who did not in the ‘After’ period (Supplementary Table S4).

This study demonstrates that culturally tailored continuity of midwifery care significantly improves perinatal outcomes for women and their First Nations babies. These findings, considered alongside other key outcomes of the overarching study—high satisfaction,^16^ strong model uptake,^13^ increased breastfeeding,^22^ and midwives’ positive experiences^18^^,^^31^—underscore the value of this model. The model is embedded and expanded at all three sites, with increasing numbers of First Nations midwives working in the models, and many First Nations midwifery students undertaking clinical placements in the model, contributing to increasing First Nations midwifery workforce.

Given the strong evidence that the Baggarrook Yurrongi programme is ‘closing the gap’ in perinatal health outcomes, we recommend model rollout, with appropriate adaptations to meet the specific needs of local First Nations communities. We recommend that policy makers provide funding to enable services to establish and deliver these models in collaboration with local communities, and that responsibility for sustaining it should no longer rest with researchers and grant funding bodies. Ongoing government investment is essential to ensure equitable, culturally safe maternity care for First Nations families.

## Contributors

DAF: Conceptualised and designed the study, data curation, formal analysis, funding acquisition, project administration and management, supervision of research staff, co-wrote original draft, reviewed and edited subsequent drafts. TS: Data curation, formal analysis, project administration and management, reviewed and edited drafts. FEMc-H: Data curation, formal analysis, project administration and management, supervision of research staff, co-wrote original draft, reviewed and edited drafts. RMcC: Contributed to study methodology, reviewed and edited drafts. GB: Contributed to project administration, and site implementation, reviewed and edited drafts. MN: Conceptualised and designed the study, data curation, funding acquisition, supervision of research staff, reviewed and edited drafts. RH: Data curation, project implementation, reviewed and edited drafts. RM: Data curation, project implementation, reviewed and edited drafts. SEJ: Conceptualised and designed the study, funding acquisition, reviewed and edited drafts. CC: Conceptualised and designed the study, funding acquisition, reviewed and edited drafts. SK: Conceptualised and designed the study, funding acquisition, reviewed and edited drafts. TS: Contributed to study methodology, reviewed and edited drafts. MJ: Conceptualised and designed the study, funding acquisition, input into project implementation, reviewed and edited drafts. JB: Conceptualised and designed the study, funding acquisition, reviewed and edited drafts. KF: Conceptualised and designed the study, reviewed and edited drafts. NB: Conceptualised and designed the study, reviewed and edited drafts. JF: Conceptualised and designed the study, funding acquisition, reviewed and edited drafts. HLMc: Conceptualised and designed the study, data curation, formal analysis, funding acquisition, project administration and management, supervision of research staff, co-wrote original draft, reviewed and edited subsequent drafts. All authors read and approved the submitted manuscript and contributed to its review and editing. DAF and TS accessed and verified the underlying data.

## Data sharing statement

De-identified dataset requests will be considered on a case-by-case basis via a data sharing agreement when the request is accompanied by a proposal found to be compliant with the ethical standards of La Trobe University.

## Declaration of interests

RMc received a Lowitja Institute post-graduate top-up scholarship for her PhD. All other authors declare no competing interests.
