# Supplementary material for: The effect of culturally tailored continuity of midwifery care on perinatal outcomes for women having a first Nations baby in Victoria, Australia: a prospective non-randomised translational study
Source: eClinicalMedicine. 2026 Jun 24;97:104028. doi: 10.1016/j.eclinm.2026.104028 (PMC13420611; doi:10.1016/j.eclinm.2026.104028)
Supplement: Supplementary Tables S1–S5 [file mmc1.docx]

**The effect of culturally tailored continuity of midwifery care on perinatal outcomes for women having a First Nations baby in Victoria, Australia: a prospective non-randomised translational study**

**SUPPLEMENTARY TABLES**

**S1. Characteristics of all women in the dataset, with mothers of First Nations babies shown by First Nations status.**

| **Characteristic** | **Before** | | | **After** | | |
| --- | --- | --- | --- | --- | --- | --- |
|  | **First Nations baby** | | | **First Nations baby** | | |
|  | **No** | **Yes** | | **No** | **Yes** | |
|  | **n (%)** | **First Nations mother** | | **n (%)** | **First Nations mother** | |
|  |  | **No**  **n (%)** | **Yes**  **n (%)** |  | **No**  **n (%)** | **Yes**  **n (%)** |
| **Age** (yrs, mean, sd) | 31.5 (5.1) | 27.3 (5.7) | 27.3 (6.1) | 32.4 (4.9) | 29.2 (6.1) | 28.4 (5.7) |
| **Partnered/married** | 87,986 (88.6) | 105 (66.9) | 556 (56.5) | 55,765 (89.7) | 187 (62.8) | 456 (62.3) |
| **Primiparous** | 47,302 (47.3) | 73 (45.9) | 397 (39.7) | 30,673 (49.1) | 132 (44.2) | 269 (36.4) |
| **Smoking < 20 wks** | 5,592 (5.6) | 49 (30.8) | 383 (38.9) | 2,437 (3.9) | 71 (23.8) | 240 (32.9) |
| **BMI** |  |  |  |  |  |  |
| <18.5 | 3,008 (3.1) | 3 (2.0) | 29 (3.2) | 1,574 (2.6) | 5 (1.8) | 28 (4.3) |
| 18.5-24.9 | 50,536 (52.6) | 73 (47.4) | 377 (41.3) | 30,528 (51.2) | 105 (38.2) | 226 (34.6) |
| 25-35 | 34,906 (36.4) | 56 (36.4) | 361 (39.6) | 22,622 (37.9) | 105 (38.2) | 250 (38.3) |
| >35 | 7,544 (7.9) | 22 (14.3) | 145 (15.9) | 4,932 (8.3) | 60 (21.8) | 149 (22.8) |

NB: denominators not provided as differing missing data across variables, but % reflect correct denominators for all outcomes.

**S2. Primary analysis – key clinical outcomes where First Nations baby’s mothers received caseload care (‘After’ period) compared to ‘Before’ period when model not in place, including ONLY those babies whose *mother was First Nations***

| **Outcome** | **First Nations mothers ‘Before’** | **First Nations**  **mothers ‘After’**  **(had caseload)** |  |  |
| --- | --- | --- | --- | --- |
|  | n (%)  (n= 999) | n (%)  (n= 399) | **OR (CI)** | **Adj* OR (CI)** |
| **Low birthweight** |  |  |  |  |
| Yes | 163 (16.3) | 57 (11.7) | 0.68 (0.49, 0.94) | 0.75 (0.52, 1.08) |
| No | 835 (83.7) | 429 (88.3) | Ref | Ref |
| **SGA^#^** |  |  |  |  |
| Yes | 21 (2.1) | 9 (1.9) | 0.88 (0.40, 1.93) | 0.94 (0.41, 2.13) |
| No | 978 (97.9) | 477 (98.2) | Ref | Ref |
| **Preterm birth** |  |  |  |  |
| Yes | 188 (18.8) | 68 (14.0) | 0.70 (0.52, 0.95) | 0.69 (0.49, 0.97) |
| No | 811 (81.2) | 418 (86.0) | Ref | Ref |
| **Admitted NICU^a^** |  |  |  |  |
| Yes | 287 (29.1) | 111 (22.9) | 0.72 (0.56, 0.93) | 0.74 (0.56, 1.98) |
| No | 698 (70.9) | 373 (77.1) | Ref | Ref |
| **Stillborn** |  |  |  |  |
| Yes | 13 (1.3) | 2 (0.4) | 0.31 (0.07, 1.39) | 0.30 (0.04, 2.54) |
| No | 986 (98.7) | 484 (99.6) | Ref | Ref |
| **Healthy infant^b^** |  |  |  |  |
| Yes | 657 (65.8) | 353 (72.6) | 1.38 (1.09, 1.75) | 1.34 (1.03, 1.75) |
| No | 342 (34.2) | 133 (27.4) | Ref | Ref |
| **Smoking >20 weeks** |  |  |  |  |
| Yes | 303 (31.9) | 131 (27.8) | 0.82 (0.64, 1.05) | 0.84 (0.64, 1.10) |
| No | 648 (68.1) | 341 (72.3) | Ref | Ref |
| **BF^c^ initiation** |  |  |  |  |
| Yes | 822 (85.5) | 435 (91.6) | 1.84 (1.27, 2.66) | 2.38 (1.52, 3.73) |
| No | 139 (14.5) | 40 (8.4) | Ref | Ref |

^#^ SGA – small for gestational age. ^a^ Neonatal special or intensive care (data not able to be separated). ^b^ Born alive, at term, of normal weight and size, and not admitted to NICU. ^c^ Breastfeeding. *Adjusted for age (continuous), BMI (group), marital status, parity, diabetes and hypertensive disorder. NB: denominators differ slightly across variables, but % reflect correct denominators for all outcomes. This analysis excluded First Nations babies whose mother was not First Nations.

**S3. Key clinical outcomes of First Nations mothers and babies compared with non-First Nations mothers and babies, showing ‘Before’ and ‘After’ outcomes (where mothers of First Nations babies received usual care ‘Before’ and tailored caseload care ‘After’), including ONLY those babies whose *mother was First Nations***

|  | **Before** | | | | **After** | | | |
| --- | --- | --- | --- | --- | --- | --- | --- | --- |
|  | **First Nations mother** | | **OR (CI)** | **Adj* OR (CI)** | **First Nations mother** | | **OR (CI)** | **Adj* OR (CI)** |
|  | **No**  n (%) | **Yes**  n (%) |  |  | **No**  n (%) | **Yes**  n (%) |  |  |
| **Low birthweight** | |  |  |  |  |  |  |  |
| Yes | 7,220 (7.2) | 163 (16.3) | 2.51 (2.12, 2.98) | 2.06 (1.70, 2.51) | 4,450 (7.1) | 57 (11.7) | 1.74 (1.32, 2.30) | 1.52 (1.11, 2.08) |
| No | 92,948 (92.8) | 835 (83.7) |  | Ref | 58,344 (92.9) | 429 (88.3) |  | Ref |
| **SGA^#^** |  |  |  |  |  |  |  |  |
| Yes | 3,481 (3.5) | 21 (2.1) | 0.60 (0.39, 0.92) | 0.62 (0.39, 0.99) | 1,543 (2.5) | 9 (1.9) | 0.75 (0.39, 1.45) | 0.92 (0.47, 1.79) |
| No | 96,733 (96.5) | 978 (97.9) |  | Ref | 61,256 (97.5) | 477 (98.2) |  | Ref |
| **Preterm birth** |  |  |  |  |  |  |  |  |
| Yes | 8,180 (8.2) | 188 (18.8) | 2.61 (2.22, 3.06) | 2.21 (1.84, 2.65) | 4,898 (7.8) | 68 (14.0) | 1.92 (1.49, 2.49) | 1.63 (1.21, 2.18) |
| No | 92,001 (91.8) | 811 (81.2) |  | Ref | 57,885 (92.2) | 418 (86.0) |  | Ref |
| **Admission to NICU^a^** | |  |  |  |  |  |  |  |
| Yes | 13,044 (13.1) | 287 (29.1) | 2.73 (2.37, 3.13) | 2.26 (1.94, 2.64) | 8,632 (13.9) | 111 (22.9) | 1.85 (1.49, 2.29) | 1.46 (1.15, 1.84) |
| No | 86,526 (86.9) | 698 (70.9) |  | Ref | 53,582 (86.1) | 373 (77.1) |  | Ref |
| **Stillborn** |  |  |  |  |  |  |  |  |
| Yes | 680 (0.7) | 13 (1.3) | 1.93 (1.11, 3.35) | 1.30 (0.61, 2.76) | 601 (1.0) | 2 (0.4) | 0.43 (0.11, 1.72) | 0.24 (0.03, 1.72) |
| No | 99,530 (99.3) | 986 (98.7) |  | Ref | 62,190 (99.0) | 484 (99.6) |  | Ref |
| **Healthy infant^b^** | |  |  |  |  |  |  |  |
| Yes | 83,271 (83.1) | 657 (65.8) | 0.39 (0.34, 0.45) | 0.49 (0.42, 0.56) | 51,722 (82.4) | 353 (72.6) | 0.57 (0.47, 0.69) | 0.72 (0.58, 0.90) |
| No | 16,943 (16.9) | 342 (34.2) |  | Ref | 11,077 (17.6) | 133 (27.4) |  | Ref |
| **Smoking >20 weeks** | |  |  |  |  |  |  |  |
| Yes | 4,338 (4.4) | 303 (31.9) | 10.21 (8.88, 11.74) | 4.73 (4.01, 5.58) | 2,154 (3.5) | 131 (27.8) | 10.75 (8.75, 13.21) | 4.75 (3.72, 6.07) |
| No | 94,747 (95.6) | 648 (68.1) |  | Ref | 60,292 (96.6) | 341 (72.3) |  | Ref |
| **BF^c^ initiation** |  |  |  |  |  |  |  |  |
| Yes | 94,495 (95.9) | 822 (85.5) | 0.25 (0.21, 0.30) | 0.49 (0.40, 0.60) | 59,060 (96.1) | 435 (91.6) | 0.45 (0.32, 0.62) | 1.10 (0.73, 1.66) |
| No | 4,023 (4.1) | 139 (14.5) |  | Ref | 2,432 (4.0) | 40 (8.4) |  | Ref |
| ^#^ SGA – small for gestational age. ^a^ Neonatal special or intensive care (data not able to be separated). ^b^ Born alive, at term, of normal weight and size, and not admitted to NICU. ^c^ Breastfeeding. *Adjusted for age (continuous), BMI (group), marital status, parity, diabetes and hypertensive disorder. NB: denominators differ slightly across variables, but % reflect correct denominators for all outcomes. This analysis excluded First Nations babies whose mother was not First Nations. | | | | | | | | |

**S4. Key clinical outcomes of First Nations mothers and babies in the ‘After’ period, comparing women who received caseload care with those who did not, including ONLY those babies whose *mother was First Nations***

|  | **Received caseload** | |  |  |
| --- | --- | --- | --- | --- |
| **Outcome** | **No**  n (%) | **Yes**  n (%) | **OR (CI)** | **Adj* OR (CI)** |
| **Low birthweight** |  |  |  |  |
| Yes | 60 (23.7) | 57 (11.7) | 0.43 (0.29, 0.64) | 0.40 (0.25, 0.64) |
| No | 193 (76.3) | 429 (88.3) | Ref | Ref |
| **SGA^#^** |  |  |  |  |
| Yes | 5 (2.0) | 9 (1.9) | 0.94 (0.31, 2.82) | 1.11 (0.29, 4.27) |
| No | 248 (98.0) | 477 (98.2) | Ref | Ref |
| **Preterm birth** |  |  |  |  |
| Yes | 59 (23.4) | 68 (14.0) | 0.53 (0.36, 0.78) | 0.54 (0.34, 0.85) |
| No | 193 (76.6) | 418 (86.0) | Ref | Ref |
| **Admitted NICU^a^** |  |  |  |  |
| Yes | 92 (38.0) | 111 (22.9) | 0.49 (0.35, 0.68) | 0.50 (0.34, 0.73) |
| No | 150 (62.0) | 373 (77.1) | Ref | Ref |
| **Stillborn** |  |  |  |  |
| Yes | 11 (4.4) | 2 (0.4) | 0.09 (0.02, 0.41) | 0.07 (0.01, 0.60) |
| No | 242 (95.7) | 484 (99.6) | Ref | Ref |
| **Healthy infant^b^** |  |  |  |  |
| Yes | 138 (54.6) | 353 (72.6) | 2.21 (1.61, 3.04) | 2.11 (1.46, 3.04) |
| No | 115 (45.5) | 133 (27.4) | Ref | Ref |
| **Smoking >20 weeks** |  |  |  |  |
| Yes | 88 (36.4) | 131 (27.8) | 0.67 (0.48, 0.94) | 0.66 (0.45, 0.97) |
| No | 154 (63.6) | 341 (72.3) | Ref | Ref |
| **BF^c^ initiation** |  |  |  |  |
| Yes | 193 (81.4) | 435 (91.6) | 2.48 (1.56, 3.93) | 3.15 (1.76, 5.63) |
| No | 44 (18.6) | 40 (8.4) | Ref | Ref |
| SGA – small for gestational age. ^a^ Neonatal special or intensive care (data not able to be separated). ^b^ Born alive, at term, of normal weight and size, and not admitted to NICU. ^c^ Breastfeeding. *Adjusted for age (continuous), BMI (group), marital status, parity, diabetes and hypertensive disorder. NB: denominators differ slightly across variables, but % reflect correct denominators for all outcomes. This analysis excluded First Nations babies whose mother was not First Nations. | | | | |

**S5. Key clinical outcomes for First Nations mothers and babies ‘Before’ and ‘After’ (regardless of whether or not caseload model received in ‘After’ period), including ONLY those babies whose *mother was First Nations***

| **Outcome** | **Before**  n (%) | **After**  n (%) | **OR (95% CI)** | **Adj* OR (95% CI)** |
| --- | --- | --- | --- | --- |
| **Low birthweight** |  |  |  |  |
| Yes | 163 (16.3) | 117 (15.8) | 0.96 (0.74, 1.25) | 1.03 (0.76, 1.40) |
| No | 835 (83.7) | 622 (84.2) |  | Ref |
| **SGA^#^** |  |  |  |  |
| Yes | 21 (2.1) | 14 (1.9) | 0.90 (0.45, 1.78) | 0.88 (0.41, 1.85) |
| No | 978 (97.9) | 725 (98.1) |  |  |
| **Preterm birth** |  |  |  |  |
| Yes | 188 (18.8) | 127 (17.2) | 0.90 (0.70, 1.15) | 0.85 (0.64, 1.14) |
| No | 811 (81.2) | 611 (82.8) |  | Ref |
| **Admitted NICU^a^** |  |  |  |  |
| Yes | 287 (29.1) | 203 (28.0) | 0.94 (0.76, 1.17) | 0.93 (0.73, 1.18) |
| No | 698 (70.9) | 523 (72.0) |  | Ref |
| **Stillborn** |  |  |  |  |
| Yes | 13 (1.3) | 13 (1.8) | 1.36 (0.63, 2.95) | 1.67 (0.55, 5.05) |
| No | 986 (98.7) | 726 (98.2) |  | Ref |
| **Healthy infant^b^** |  |  |  |  |
| Yes | 657 (65.8) | 491 (66.4) | 1.03 (0.84, 1.26) | 1.05 (0.84, 1.32) |
| No | 342 (34.2) | 248 (33.6) |  | Ref |
| **Smoking >20 weeks** |  |  |  |  |
| Yes | 303 (31.9) | 219 (30.7) | 0.95 (0.77, 1.17) | 0.97 (0.76, 1.23) |
| No | 648 (68.1) | 495 (69.3) |  | Ref |
| **BF^c^ initiation** |  |  |  |  |
| Yes | 822 (85.5) | 628 (88.2) | 1.26 (0.95, 1.69) | 1.53 (1.08, 2.17) |
| No | 139 (14.5) | 84 (11.8) |  | Ref |

^#^ SGA – small for gestational age. ^a^ Neonatal special or intensive care (data not able to be separated). ^b^ Born alive, at term, of normal weight and size, and not admitted to NICU. ^c^ Breastfeeding. *Adjusted for age (continuous), BMI (group), marital status, parity, diabetes and hypertensive disorder. NB: denominators differ slightly across variables, but % reflect correct denominators for all outcomes. This analysis excluded First Nations babies whose mother was not First Nations.
